# Time Gating of Chloroplast Autofluorescence Allows Clearer Fluorescence Imaging *In Planta*

**DOI:** 10.1371/journal.pone.0152484

**Published:** 2016-03-30

**Authors:** Yutaka Kodama

**Affiliations:** Center for Bioscience Research and Education, Utsunomiya University, Tochigi, 321–8505, Japan; Pennsylvania State Hershey College of Medicine, UNITED STATES

## Abstract

Chloroplast, an organelle facilitating photosynthesis, exhibits strong autofluorescence, which is an undesired background signal that restricts imaging experiments with exogenous fluorophore in plants. In this study, the autofluorescence was characterized *in planta* under confocal laser microscopy, and it was found that the time-gated imaging technique completely eliminates the autofluorescence. As a demonstration of the technique, a clearer signal of fluorescent protein-tagged phototropin, a blue-light photoreceptor localized at the chloroplast periphery, was visualized *in planta*.

## Introduction

The chloroplast, which is an organelle facilitating photosynthesis, contains many pigments such as chlorophylls and carotenoids localized at the thylakoid membrane for light harvesting. It is well known that chloroplasts exhibit very strong autofluorescence in red, with a peak of approximately 680 nm [1]. A major pigment contributing to autofluorescence is chlorophyll, and energy transfer from carotenoids to chlorophyll also contributes to the rise. The autofluorescence is important for re-absorbance by chlorophyll to maximize photosynthetic activity. In fluorescence imaging experiments using exogenous fluorophore such as fluorescent dyes and proteins, the autofluorescence is considered to be an undesired background signal in photosynthetic organisms such as plants under fluorescent and/or confocal laser microscopy [2]. A previous study reported the interference of chlorophyll autofluorescence with green fluorescent protein (GFP) [2]. To eliminate the autofluorescence so as to obtain clearer fluorescence imaging of GFP, ethanol, herbicides, etiolation or gene silencing has been applied to remove pigments *in planta* [2–4]. However, because these eliminating strategies result in an abnormal physiological condition, a new strategy available under the physiological condition is necessary.

In this study, autofluorescence of chloroplast is characterized by using confocal laser microscopy, and it is revealed that time-gated fluorescence imaging technique completely eliminates the autofluorescence in fluorescence imaging experiments using exogenous fluorophore *in planta*.

## Materials and Methods

### Plant material and growth conditions

Male thalli of the liverwort *Marchantia polymorpha*, accession Takaragaike-1 (Tak-1), and transgenic liverworts, Citrine line (FM4) [5] and Mpphot-Citrine line (see below), were grown in a culture room (temperature: 22°C, humidity: approximately 40%) and were asexually maintained on half strength Gamborg’s B5 (1/2 B5) medium [6] with 1% agar under 75 μmol photons m^−2^ s^−1^ continuous white light [5,7]. For observation, one-day-old gemmalings (immature thalli grown from gemmae) obtained from approximately 1-month-old thalli of Tak-1 were used [8]. *Arabidopsis thaliana* was grown in soil for 3 weeks at approximately 100 μmol m^−2^ s^−1^ under a 16-h/8-h light/dark cycle at 23°C in a growth chamber, and the leaves were detached for observation [9].

### Plasmid construction

To construct a gene for Citrine-fused Mpphot (Mpphot-Citrine), the binary vector pMpGWB106 for *Agrobacterium*-mediated transformation of *M*. *polymorpha* was used [8,10]. The pMpGWB106 harbors *Citrine* gene to fuse at the 3′-terminus of the target gene, and it is adapted to the gateway cloning system (Invitrogen, CA, USA). The coding sequence (3,345 bp) of the Mp*PHOT* gene (accession number: AB938188) was amplified from the cDNA library of *M*. *polymorpha* by polymerase chain reaction (PCR) with the following primers: 5′-GGGGACAAGTTTGTACAAAAAAGCAGGCTTCATGATGCCCTCCACGGAT-3′ and 5′-GGGGACCACTTTGTACAAGAAAGCTGGGTCATATTCATCAAATGAGGCG-3′. The amplified DNA fragments were cloned into a pDONR207 plasmid by BP reaction (Invitrogen, CA, USA). The resulting plasmid (pDONR207-MpPHOT) was mixed with pMpGWB106, and the LR reaction (Invitrogen, CA, USA) was performed to generate pMpGWB106-MpPHOT, which contains a fusion gene for Mpphot-Citrine.

### *Agrobacterium*-mediated transformation of *M*. *polymorpha*

*Agrobacterium*-mediated transformation of *M*. *polymorpha* was performed by the AgarTrap method using sporelings [11]. The resulting transformants of *M*. *polymorpha* (T1) were cultivated for approximately 1 month, and then gemmae (G1) were obtained. After assessing sex by PCR [11], a male G1 gemmaling was selected and designated the Mpphot-Citrine-2-1 line. The G1 gemma was cultivated for 1 month, and then gemmae (G2) were obtained. For observation, 1-day-old transgenic G2 gemmalings were used.

### Confocal laser microscopy

Confocal laser microscopy (Leica TCS SP8X) equipped with a hybrid detector (commercially, HyD^TM^) and a highly flexible pulsed white-light laser (commercially, WLL) was used, according to the instructions of the manufacture (Leica Microsystemes, Wetzlar, Germany). As commonly used, the Leica Application Suite X (LAS X) was used as a software platform, the objective lens used was the HC PL APO CS 63 × 1.20 WATER, scan speed was 400 Hz (400 lines/s) at a resolution at 512 × 512 pixels, and normal acquisition of the hybrid detector (standard mode of HyD^TM^) was used. The emission spectra of chloroplast autofluorescence were measured with 470-nm excitation laser from the white-light laser, and xyλ scanning was performed using the hybrid detector at 478–739 nm wavelength region. Excitation spectra of chloroplast autofluorescence were measured with an emission at 680–700 nm, and xyΛ scanning was performed with the white-light laser at 470–626 nm wavelength region. To scan spectra, neither line nor frame averaging was used. To capture the fluorescent images, the hybrid detector was used at the indicated green–yellow wavelength region, and the conventional photomultiplier tube (PMT) was used at the indicated red wavelength region. For imaging at green and yellow wavelength regions, 488-nm laser (35% of white-light laser; approximately 7.3 μW) and 514-nm laser (35% of white-light laser; approximately 8.8 μW) were employed, respectively. The laser power after passing through the objective lens was measured by using a power meter 1918-R (Newport Corporation, CA, USA) with a silicon detector 918D-SL-OD1 (detector active area: 1 cm^2^) (Newport Corporation, CA, USA). To capture bright field images, PMT for transmittance was used (Scan-BF mode of PMT trans in LAS X software). For the capture of the images, line and frame averages used were four and two times, respectively.

## Results and Discussion

**Fig 1:**
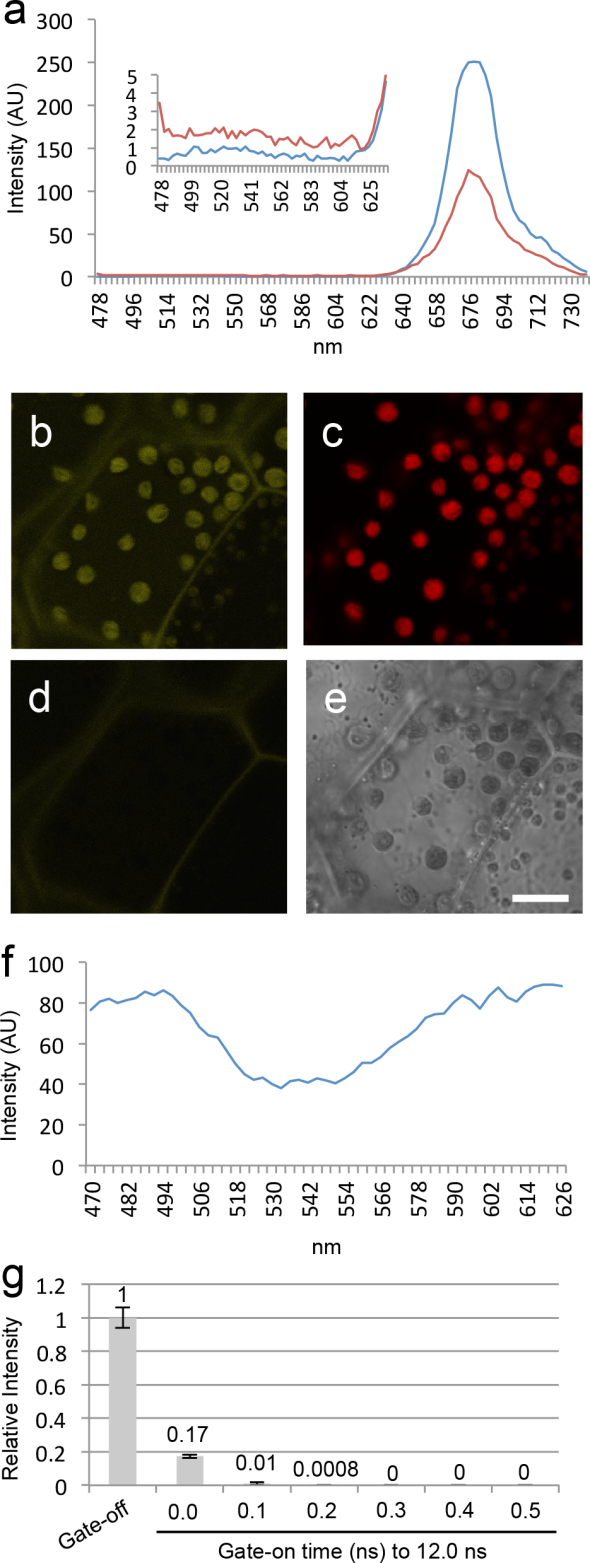
Chloroplast autofluorescence and time-gated rejection in *Marchantia polymorpha*. Chloroplast autofluorescence (a) emission spectra from unbleached (blue) and bleached (red) chloroplasts. Light intensities as arbitrary unit (AU). (b-d) Representative images at the yellow wavelength region (520–561 nm) (b), at the red wavelength region (648–709 nm) (c), and at the yellow wavelength region (520–561 nm) with time gating (gate-on time: 0.3–12.0 ns) (d). (e) Bright field image. Scale bar, 10 μm. (f) Excitation spectrum of chloroplast autofluorescence. (g) Effects of time-gated fluorescence imaging technique at the yellow wavelength region. Relative light intensities are shown. Experiments were performed thrice (with three different samples); bars represent standard deviations. The averaged number is shown at the each graph.

To explore a new strategy for the elimination of chloroplast autofluorescence in fluorescence imaging experiments, the autofluorescence was firstly characterized in *M*. *polymorpha*. For this, confocal laser microscopy was used, using the Leica TCS SP8X equipped with a hybrid detector and a highly flexible pulsed white-light laser. The measurement of emission spectra with a 470-nm excitation laser revealed fluorescence with a peak at 680 nm (Fig 1A) and a broad range spectrum (Fig 1A, the inside graph). In fact, the autofluorescence was detected not only in the red wavelength region (648–709 nm) but also in the yellow wavelength region (520–561 nm) (Fig 1B, 1C and 1E). For example, it has been determined that when fluorescent proteins, including green (GFP) and yellow (YFP) are used, the autofluorescence at the shorter wavelength region hinders the fluorescence imaging in plants [2]. As observations that are never reported, it appears that many researchers experience an increase of chloroplast autofluorescence at the green wavelength region of emission during the observation of GFP with a 488-nm laser in plants, and the current study verified this phenomenon. After chloroplasts were irradiated by a 488-nm laser (70% of white-light laser; approximately 14.3 μW) for 30 s to bleach chlorophyll, the emission spectrum was measured with 470-nm laser. Results showed that the spectral peak at 680 nm decreased (Fig 1A), whereas fluorescence in the shorter wavelength region (approximately 480–620 nm) was increased (Fig 1A, the inside graph). Although the fluorescence increased in the shorter wavelength region might be originated from photoconversion of unknown pigment(s) or reduction of energy transfer to chlorophyll from other pigments such as carotenoids, it remains to be determined. In addition, when the excitation spectrum was measured with an emission at 680–700 nm under the confocal laser microscopy, all laser lines excited the autofluorescence (Fig 1F). Ironically, the 488-nm laser that is widely used for fluorescence imaging mostly excites the autofluorescence (Fig 1F), indicating that it is inappropriate for fluorescence imaging in plants. In addition, although the spectrum in fluorescence imaging experiments is normally employed by the selection of emission and excitation wavelengths, my characterization of the autofluorescence suggested that elimination of the autofluorescence is difficult when only using selections of emission and excitation wavelengths.

Next, it was focused on a time-gated fluorescence imaging, which is a time-resolved fluorescence detection method with lifetime of fluorescence [12]. A combination of a hybrid detector with a pulsed white-light laser in the confocal laser microscopy facilitates the use of the system for time-gated fluorescence imaging (commercially known as LightGate in Leica Microsystems). The white-light laser system is a pulsed laser with a 12.5 ns time interval, and the detection time of the hybrid detector can be altered in a range of 0.0–12.0 ns. In time-gated fluorescence imaging, short-lifetime fluorescence is decayed during time interval of pulsed laser, and only long-lifetime fluorescence can be captured at a delayed time after decline of short-lifetime of fluorescence. As the lifetime of chlorophyll fluorescence is known at a picoseconds level [1], it was predicted that chloroplast autofluorescence is eliminated by the system *in planta*. To date, the time-gated fluorescence imaging technique has not been applied to eliminate chloroplast autofluorescence. When the detection time of the hybrid detector was restricted during 0.0–12.0 ns, chloroplast autofluorescence induced by a 514-nm laser at a yellow wavelength region (520–561 nm) was reduced by 17% (Fig 1G). When a detection time-gated with 0.3–12.0 ns was used, it completely eliminated the autofluorescence; i.e., no autofluorescence of chloroplast was achieved under a normal physiological condition (Fig 1D and 1G). A similar result was found in the autofluorescence induced by a 488-nm laser at a green wavelength region (495–535 nm) for GFP imaging (S1 Fig). In addition, the time-gated rejection of chloroplast autofluorescence was achieved in a vascular model plant *A*. *thaliana* (S2 Fig), indicating that the technique can be applied to various plant species.

**Fig 2:**
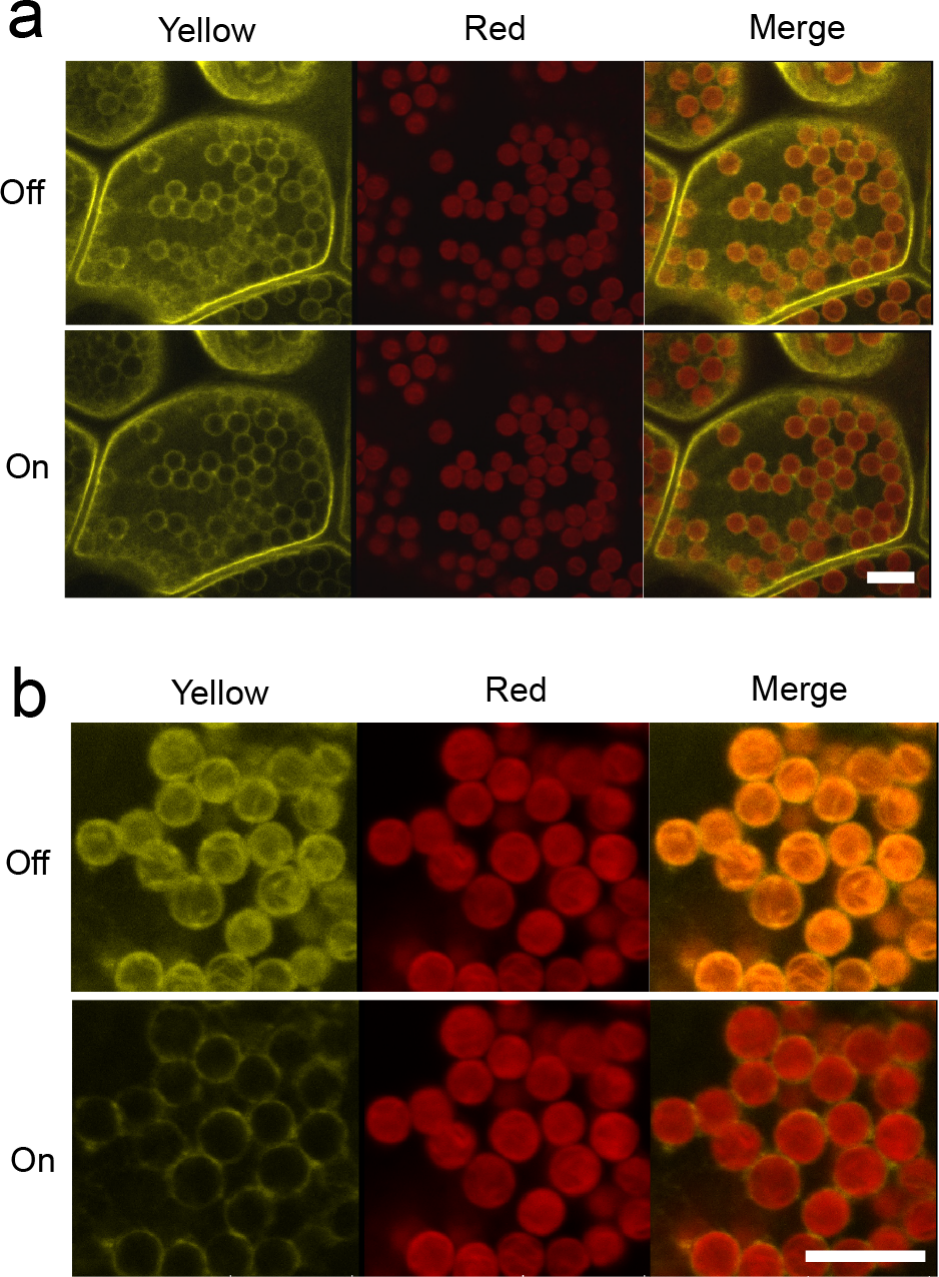
Fluorescence imaging of Mpphot-Citrine on the chloroplast periphery of transgenic *Marchantia polymorpha*. (a) Single cellular fluorescence imaging. (b) Intracellular fluorescence imaging. Upper and lower panels reveal gate-off and gate-on (time: 0.3–12.0 ns) images, respectively. Left and center panels show images at the yellow (520–561 nm) and red (648–709 nm) wavelength regions, respectively. The right panels demonstrate merged images comprising yellow and red images. Scale bar, 10 μm.

To demonstrate the practical use of the time-gated rejection of chloroplast autofluorescence, the fluorescent signal around the chloroplast was visualized. As a model experiment, an improved YFP (Citrine)-tagged phototropin (phot), a blue-light photoreceptor localized at the chloroplast outer envelope membrane, was analyzed in *M*. *polymorpha*. Note that the lifetime of Citrine fluorescence is approximately 3.6 ns [13], and only 10% of Citrine fluorescence was reduced by the time gating with 0.3–12.0 ns (S3 Fig). Previous studies on *A*. *thaliana* reported that phot localizes on the plasma membrane in darkness, whereas some fractions translocate into the cytosol or Golgi apparatus in response to blue light [14,15]. Recently, it was also reported that phot1 and phot2 localize on the chloroplast outer envelope membrane in *A*. *thaliana* [16]. However, in other plant species, the presence of phot on the chloroplast outer envelope membrane remains to be determined. In *M*. *polymorpha*, phot is encoded by a single copy gene, which is termed “Mpphot” [17]. A previous study reported only the localization of Mpphot-Citrine at the plasma membrane [17]. In the current study, transgenic liverwort expressing Mpphot-Citrine was produced by the AgarTrap method, a genetic transformation method for *M*. *polymorpha* [11]. Confocal laser microscopic analysis determined that Mpphot-Citrine localizes not only on the plasma membrane but also on the chloroplast periphery (Fig 2A, upper panels). Using the time-gated fluorescence imaging technique with a 514-nm laser, the Citrine fluorescent signal at the chloroplast periphery was clearly visualized, with no chloroplast autofluorescence at a yellow wavelength region (520–561 nm) (Fig 2A, lower panels). When chloroplasts were scanned with a zoom, the autofluorescence at the yellow wavelength region was significantly increased, highly compromising the interpretation of the image data (Fig 2B, upper panels). When the time-gated technique was used, the autofluorescence was completely eliminated, thereby allowing a clearer visualization of Citrine fluorescence at the chloroplast periphery (Fig 2B, lower panels). The observations suggested the presence of Mpphot-Citrine on the chloroplast outer envelope membrane of *M*. *polymorpha*.

The present study characterized chloroplast autofluorescence in view of fluorescence imaging study using confocal laser microscopy and suggested that complete elimination of the autofluorescence *in planta* is difficult when only using selections of emission and excitation wavelengths. In addition, it was revealed that the time-gated fluorescence imaging technique completely eliminates chloroplast autofluorescence. Time-gated fluorescence imaging thereby facilitates clearer fluorescence imaging of Mpphot-Citrine on the chloroplast periphery under a normal physiological condition. In addition to application in plants, the rejection of autofluorescence using time-gated fluorescence imaging may be a powerful technique for fluorescence imaging with fluorescent protein, fluorescent dye or immunofluorescence in various organisms and cell types, which exhibit natural and/or artificial autofluorescence.

## Supporting Information

S1 Fig Time-gated rejection of chloroplast autofluorescence at the green wavelength region in *Marchantia polymorpha*. (a) Effects of time-gated fluorescence imaging technique at the green wavelength region (495–535 nm) using 488-nm laser. Light intensities are shown as relative intensity that was calculated by dividing the mean intensities of gate-off experiments. All experiments were performed thrice (with three different samples); bars represent standard deviations. (b) Representative images of chloroplast autofluorescence time gating at the green wavelength region. G (OFF), chloroplast autofluorescence at the green wavelength region; G (ON), chloroplast autofluorescence time gating (gate-on time: 0.3–12.0 ns) at the green wavelength region; R, chloroplast autofluorescence at the red wavelength region (648–709 nm); BF, bright field. Scale bar, 10 μm.
(PDF)

S2 Fig Time gating of chloroplast autofluorescence in *Arabidopsis thaliana*. (a) Representative images of time gating of chloroplast autofluorescence at the yellow wavelength region (520–561 nm) using a 514-nm laser. (b) Representative images of time gating of chloroplast autofluorescence at the green wavelength region (495–535 nm) using 488-nm laser. Time gating of chloroplast autofluorescence was performed at 0.3–12.0 ns as gate-on time. Chloroplast autofluorescence at the red wavelength region (648–709 nm) is shown as controls. Scale bar, 10 μm.
(PDF)

S3 Fig Observation of time-gated reduction of Citrine fluorescence intensity using rhizoid cells of *Marchantia polymorpha*. (a) Representative images of time-gated reduction of Citrine fluorescence intensity at the yellow wavelength region (520–561 nm) using a 514-nm laser. To clearly determine Citrine fluorescence, rhizoid cells were used, in which any mature chloroplast is not developed. Scale bar, 10 μm. (b) Light intensities are shown as relative intensity that was calculated by dividing the mean intensities of gate-off experiments. All experiments were performed thrice (with three different samples).
(PDF)
