# Supplementary material for: Time Gating of Chloroplast Autofluorescence Allows Clearer Fluorescence Imaging In Planta
Source: PLoS One. 2016 Mar 30;11(3):e0152484. doi: 10.1371/journal.pone.0152484 (PMC4814121; doi:10.1371/journal.pone.0152484)
Supplement: S1 Fig — (a) Effects of time-gated fluorescence imaging technique at the green wavelength region (495–535 nm) using 488-nm laser. Light intensities are shown as relative intensity that was calculated by dividing the mean intensities of gate-off experiments. All experiments were performed thrice (with three different samples); bars represent standard deviations. (b) Representative images of chloroplast autofluorescence time gating at the green wavelength region. G (OFF), chloroplast autofluorescence at the green wavelength region; G (ON), chloroplast autofluorescence time gating (gate-on time: 0.3–12.0 ns) at the green wavelength region; R, chloroplast autofluorescence at the red wavelength region (648–709 nm); BF, bright field. Scale bar, 10 μm. (PDF) [file pone.0152484.s001.pdf]

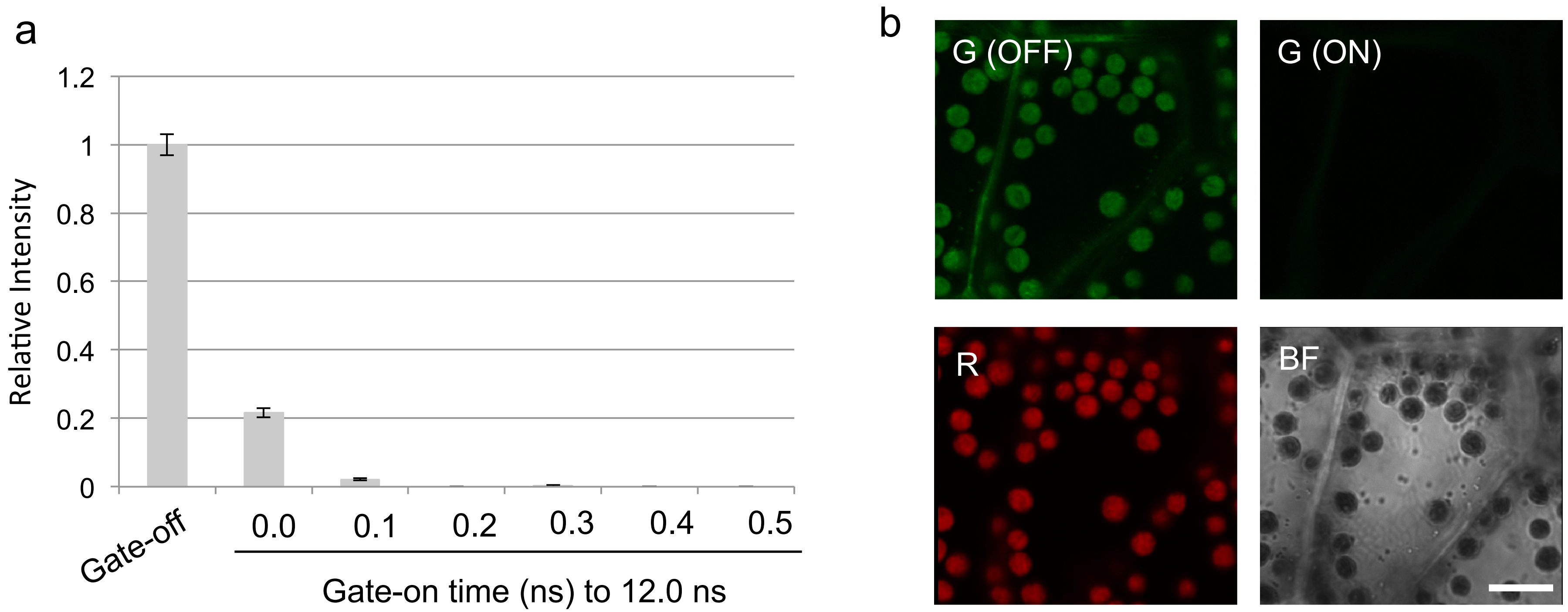

S1 Fig. Time-gated rejection of chloroplast autofluorescence at the green wavelength region in *Marchantia polymorpha*. (a) Effects of time-gated fluorescence imaging technique at the green wavelength region (495–535 nm) using 488-nm laser. Light intensities are shown as relative intensity that was calculated by dividing the mean intensities of gate-off experiments. All experiments were performed thrice (with three different samples); bars represent standard deviations. (b) Representative images of chloroplast autofluorescence time gating at the green wavelength region. G (OFF), chloroplast autofluorescence at the green wavelength region; G (ON), chloroplast autofluorescence time gating (gate-on time: 0.3–12.0 ns) at the green wavelength region; R, chloroplast autofluorescence at the red wavelength region (648–709 nm); BF, bright field. Scale bar, 10 µm.
