# Supplementary material for: Time Gating of Chloroplast Autofluorescence Allows Clearer Fluorescence Imaging In Planta
Source: PLoS One. 2016 Mar 30;11(3):e0152484. doi: 10.1371/journal.pone.0152484 (PMC4814121; doi:10.1371/journal.pone.0152484)
Supplement: S2 Fig — (a) Representative images of time gating of chloroplast autofluorescence at the yellow wavelength region (520–561 nm) using a 514-nm laser. (b) Representative images of time gating of chloroplast autofluorescence at the green wavelength region (495–535 nm) using 488-nm laser. Time gating of chloroplast autofluorescence was performed at 0.3–12.0 ns as gate-on time. Chloroplast autofluorescence at the red wavelength region (648–709 nm) is shown as controls. Scale bar, 10 μm. (PDF) [file pone.0152484.s002.pdf]

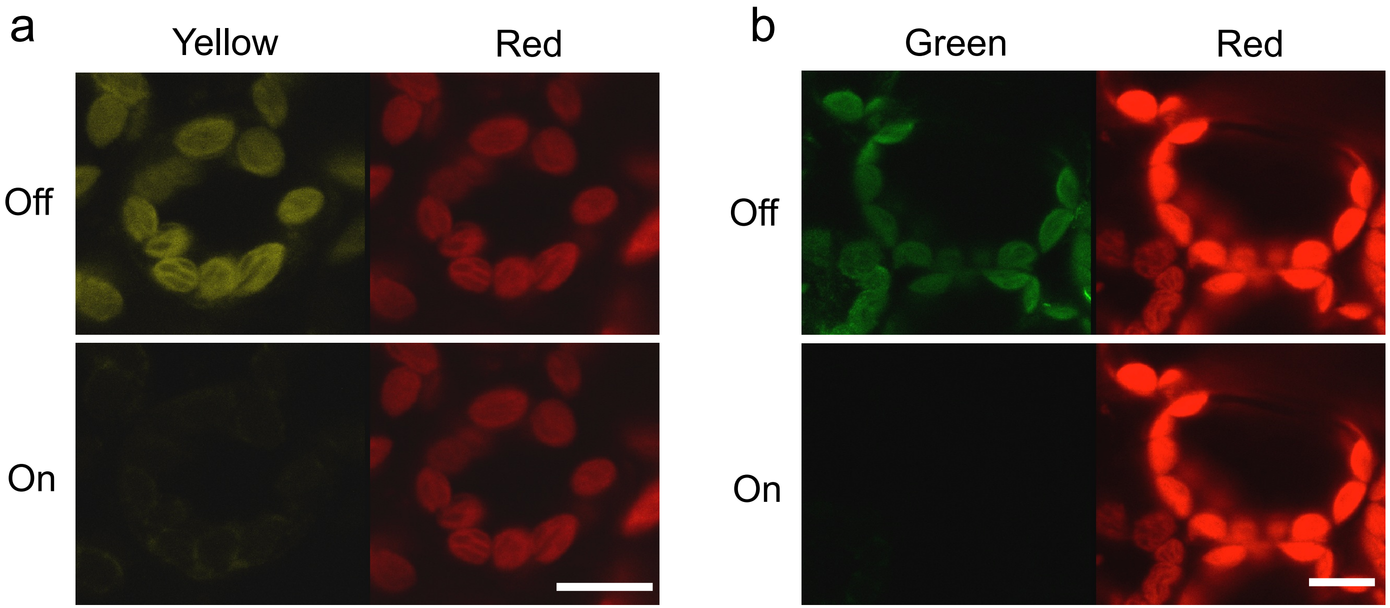

S2 Fig. Time gating of chloroplast autofluorescence in *Arabidopsis thaliana*. (a) Representative images of time gating of chloroplast autofluorescence at the yellow wavelength region (520–561 nm) using a 514-nm laser. (b) Representative images of time gating of chloroplast autofluorescence at the green wavelength region (495–535 nm) using 488-nm laser. Time gating of chloroplast autofluorescence was performed at 0.3–12.0 ns as gate-on time. Chloroplast autofluorescence at the red wavelength region (648–709 nm) is shown as controls. Scale bar, 10 µm.
