# Supplementary material for: Time Gating of Chloroplast Autofluorescence Allows Clearer Fluorescence Imaging In Planta
Source: PLoS One. 2016 Mar 30;11(3):e0152484. doi: 10.1371/journal.pone.0152484 (PMC4814121; doi:10.1371/journal.pone.0152484)
Supplement: S3 Fig — (a) Representative images of time-gated reduction of Citrine fluorescence intensity at the yellow wavelength region (520–561 nm) using a 514-nm laser. To clearly determine Citrine fluorescence, rhizoid cells were used, in which any mature chloroplast is not developed. Scale bar, 10 μm. (b) Light intensities are shown as relative intensity that was calculated by dividing the mean intensities of gate-off experiments. All experiments were performed thrice (with three different samples). (PDF) [file pone.0152484.s003.pdf]

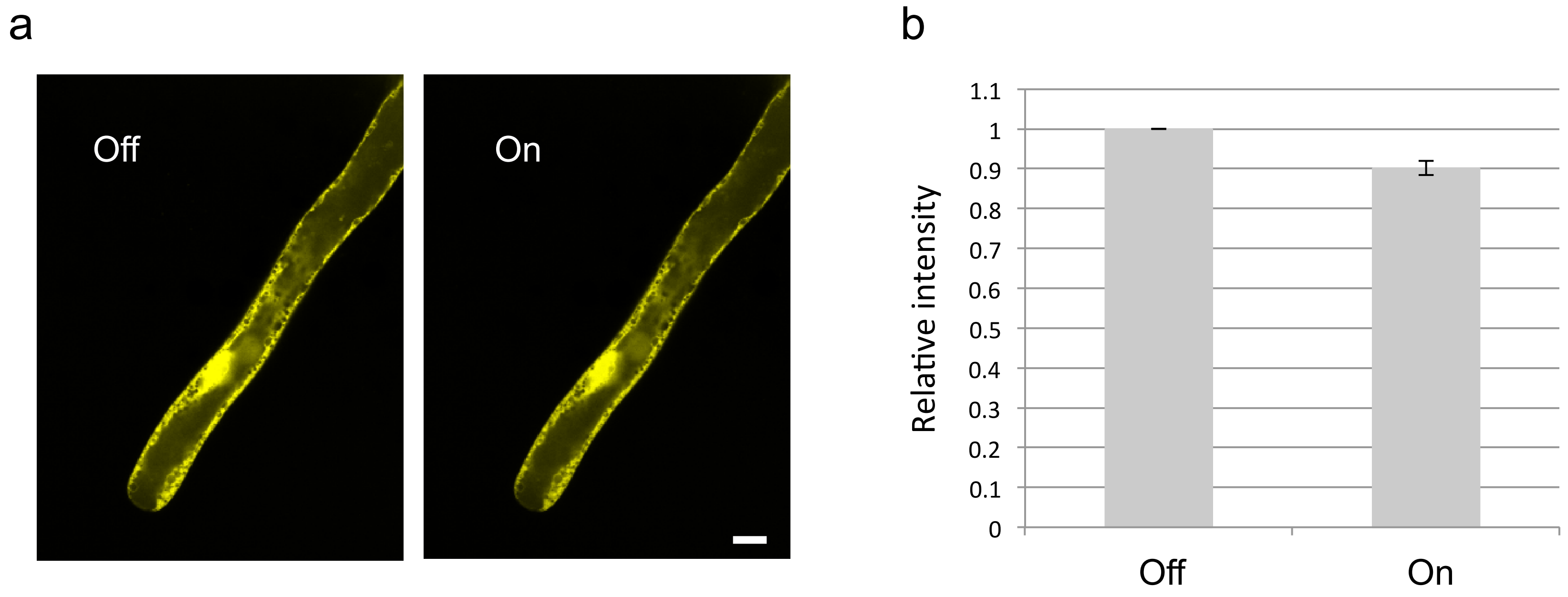

S3 Fig. Observation of time-gated reduction of Citrine fluorescence intensity using rhizoid cells of *Marchantia polymorpha*.

(a) Representative images of time-gated reduction of Citrine fluorescence intensity at the yellow wavelength region (520–561 nm) using a 514-nm laser. Time gating was performed at 0.3–12.0 ns as gate-on time. To clearly determine Citrine fluorescence, rhizoid cells were used, in which any mature chloroplast is not developed. Scale bar, 10 µm. (b) Light intensities are shown as relative intensity that was calculated by dividing the mean intensities of gate-off experiments. All experiments were performed thrice (with three different samples).
